# Genetic Diversity of *Bubalus bubalis* in Germany and Global Relations of Its Genetic Background

**DOI:** 10.3389/fgene.2020.610353

**Published:** 2021-01-22

**Authors:** Antonia Noce, Saber Qanbari, Rayner González-Prendes, Julia Brenmoehl, María Gracia Luigi-Sierra, Michael Theerkorn, Marc-André Fiege, Heike Pilz, Adrian Bota, Livia Vidu, Csaba Horwath, László Haraszthy, Pencho Penchev, Yordanka Ilieva, Tzonka Peeva, Wolfgang Lüpcke, René Krawczynski, Klaus Wimmers, Manfred Thiele, Andreas Hoeflich

**Affiliations:** ^1^Leibniz-Institute for Farm Animal Biology, Dummerstorf, Germany; ^2^Animal Breeding and Genomics Group, Wageningen University & Research, Wageningen, Netherland; ^3^Department of Animal Genetics, Center for Research in Agricultural Genomics, Campus Universitat Autonoma de Barcelona, Bellaterra, Spain; ^4^Hof am Meer GmBH, Stadland, Germany; ^5^Gut Darss, GmbH & Co. KG, Born, Germany; ^6^Wiesenburger Land eG, Wiesenburg, Germany; ^7^Research and Development Station for Buffalos Şercaia, Şercaia, Romania; ^8^University of Agronomic Sciences and Veterinary Medicine of Bucharest, Bucharest, Romania; ^9^Comuna Baciu sat Mera, Cluj, Romania; ^10^Pro Vértes, Csákvár, Hungary; ^11^Bulgarian National Association for Development of Buffalo Breeding, Shumen, Bulgaria; ^12^Higher School in Agribusiness and Development of Regions, Agricultural University Plovdiv, Tarnovo, Bulgaria; ^13^Energiequelle GmbH, Zossen, Germany; ^14^German Buffalo Association, Penig, Germany

**Keywords:** *Bubalus bubalis*, European populations, genotyping, genetic diversity, population structure, run of homozygosity

## Abstract

This is the first study to explore the genetic diversity and population structure of domestic water buffalo (*Bubalus bubalis*) in Germany and their potential relations to herds in other parts of Europe or worldwide. To this end, animals from different herds in Germany, Bulgaria, Romania, and Hungary were genotyped and compared to genotypes from other populations with worldwide distribution and open to the public. The pilot study analyzed population structure, phylogenetic tree, and inbreeding events in our samples. In buffalos from Germany, a mixed genetic make-up with contributions from Bulgaria (Murrah breed), Romania, and Italy was found. All in all, a high degree of genetic diversity was identified in European buffalos, and a novel genotype was described in Hungarian buffalos by this study. We demonstrate that European buffalos stand out from other buffalo populations worldwide, supporting the idea that buffalos have not completely disappeared from the European continent during the late Pleistocene. The high genetic diversity in European buffalos seems to be an excellent prerequisite for the establishment of local breeds characterized by unique traits and features. This study may also be considered as an initial step on the way to genome characterization for the sustainable development of the buffalo economy in Germany and other parts of Europe in the future.

## Introduction

Water buffalo (*Bubalus bubalis*) is a multipurpose animal, producing meat, milk, leather products, and dung. In developing countries, buffalos are used as draught animals, providing more than 40% of farm labor (Borghese, 2005). Due to their wallowing behavior, buffalos are less susceptible to ectoparasites and related diseases and suitable for grazing in the swamps (Michelizzi et al., 2010). Buffalos are also more efficient in the digestion process due to their longer rumen and produce a lower average of methane emission (Moss et al., 2000; Kawashima et al., 2006; Calabró et al., 2013). Compared to cattle, the buffalo is less selective for feed quality (Michelizzi et al., 2010) and longer living, granting husbandry at lower costs and with less frequent animal turnover (Borghese, 2005; Hegde, 2019; Hoffman and Valencak, 2020). Buffalo meat is a rich source of proteins, fatty acids as omega 3-6 fatty acids, iron, and characterized by lower concentrations of cholesterol if compared to cattle (Roth and Myers, 2004). Milk from buffalo contains biliverdin, bioactive pentasaccharides, and gangliosides, which are not present in bovine milk (Abd El-Salam and El-Shibiny, 2011) and more fat in total but lower cholesterol compared to the cattle. Buffalo milk is further characterized by higher protein content and big casein micelles; both features are related to the higher yields of cheese produced from the same amounts of buffalo versus dairy cow milk (Zicarelli, 2004; Martini et al., 2018).

The domestic water buffalo is classified into two major categories: swamp buffalo (*Bubalus bubalis carabanensis*, 2n = 48) and river buffalo (*Bubalus bubalis bubali*, 2n = 50). While their taxonomical status is still being debated, *B. bubalis* is supposed to be descended from Indian wild buffalo (*Bubalus arnee*) domesticated ≈5,000 years ago (Cockrill, 1984). Nevertheless, the origin of the two subspecies into which water buffalo are divided is still the object of study (Colli et al., 2018b), especially to solve the debate about the occurrence of a single versus two separate domestication events (Lau et al., 1998; Kierstein et al., 2004; Kumar et al., 2007).

Little is known about the history, genetic diversity, and performance of European buffalo populations. The presence of water buffalo in Europe is dated from the Pleistocene until the warm period before the last Eem-Interglacial (≈125,000 years ago), when buffalos were also present in Central Europe (Zahariev et al., 1986; Alexiev, 1998). Although hunting (Martin, 1984) and climatic changes, particularly during the late Pleistocene (Lima-Ribeiro and Diniz-Filho, 2017), temporarily or locally displaced and shrank the European buffalo populations, the presence of refugial regions in Southern and Eastern Europe may have prevented the extinction of the *Bubalus* species from the European continent (Krawczynski, 2010). The presence of buffalos in South-Eastern Europe during the early Neolithikum (9,000–7,000 BC) was suggested (Bökönyi, 1957) but generally dismissed (Bartosiewicz, 1999). Based on bone finds in Austria dated to the Atlantikum (7,000–4,000 BC), other authors have also discussed the presence of buffalos during the Holocene (beginning 12,000 BC) (Pucher, 1988). However, defined criteria for the unambiguous distinction of members from the Bovini tribus (in particular, *Bos* versus *Bubalus*) have been lacking until now (Pucher, 1988). According to Haarmann (2011), the ancient Greeks have used different terms for aurochs (*Bos primigenius*), wisent (*Bos bonasus*), and buffalo (*Bubalus* sp.). This linguistic approach supports the hitherto controversial bone finds from the same area at about the same time. It is unclear whether water buffalos repopulated Europe after the ice age or whether they survived in refugial areas. Because rock reliefs, dated back to ≈16,000 BC in France, clearly display buffalos (Krawczynski, 2013). This, in fact, would be an indication that water buffalos in Europe survived the ice age.

Domesticated water buffalos in Europe are referenced during the centuries VI–XII C.E. (Maymone, 1942; Alexiev, 1998) and had been settled in Italy, Bulgaria, Romania, and all the other Balkan countries, where they have been preserved and bred for centuries until today (Ferrara, 1964). European buffalos are river type (Borghese, 2011). The Balkans (East Europe) are an essential point of the river buffalo’s historical migration route. Riverine buffalo moved to Southwestern Asia from the Indian domestication area, reached Egypt and Turkey, and arrived in East Europe and Italy during the seventh century (Zeuner, 1963; Clutton-Brock, 1999). Some animals likely returned then to Egypt, Turkey, and Bulgaria with Crusaders returning, and spread into the other Balkan regions during the 12th century (Ferrara, 1964; Borghese, 2005). Since 1980 buffalos have also been introduced to Germany, France, Spain, Portugal, Luxembourg, The Netherlands, Switzerland, and other parts of Europe. Nowadays, the only officially recognized breeds in Europe are the Mediterranean Italian buffalo and the Bulgarian Murrah. Selecting animals for milk traits was initiated in Italy. Crossing with other populations was avoided; a herd book with animals, performance, and morphological traits was established to maintain its unique genetic identity (Iannuzzi and Di Meo, 2009). The primary purpose was the production and marketing of milk and milk derivatives such as mozzarella cheese, one of the “pasta filata” cheeses known worldwide (Zicarelli, 2001).

While local Bulgarian buffalos were a significant European Mediterranean population raised for draft power, meat, and milk purposes, they started to be crossed with Murrah breed since 1972, mainly to improve their performance for milk production. A selection program was initiated to develop a typical Bulgarian Murrah breed for milk with high butterfat content (Borghese, 2005; Borghese and Moioli, 2016).

In Germany, 7,614 buffalos, spread over 16 different regions, are recorded for the year 2020 by an internal communication from the German Buffalo Breeder’s Association. The development of the single nucleotide polymorphism (SNP) genotyping assay specific for river buffalo (Iamartino et al., 2017), Axiom^®^ Buffalo Genotyping Array 90K from Affymetrix, now enables genome analysis of this comparably novel farm animal species and could be useful for the initiation of genetic selection in German buffalo. A subsequent release of the first assembly of the water buffalo genome (Low et al., 2019) further enabled researchers to expand their knowledge about this on a molecular scale. In this study, we sought to explore the genetic diversity and structure of domestic water buffalo populations in Germany and Eastern Europe.

## Materials and Methods

### Ethics Statement

All sampling occurred as part of commercial buffalo breeding programs and strictly adhered to national and international laws.

### Samples Collection, DNA Purification, and Genotyping

**FIGURE 1:**
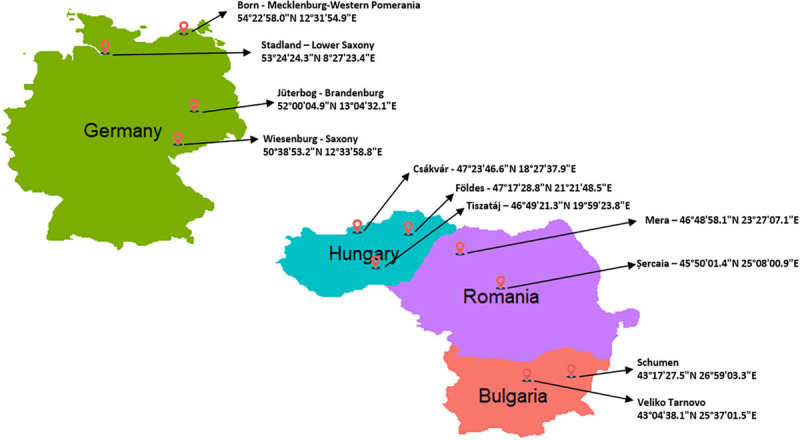
Geographic map showing the locations with specific coordinates the buffalo samples were collected from, genotyped in the current study. The number of samples for each location are reported in Table 1.

Ear tag test samples from 285 female and male buffalos were collected randomly from ten farms during the years 2018 and 2019. Importantly, all breeders were asked to provide samples from unrelated animals. The farms are distributed in Central and Eastern Europe (Figure 1), as listed in Table 1. DNeasy Blood & Tissue Kit (Qiagen, Germany) was used to extract genomic DNA according to the instructions of the manufacturer. Purification and concentration of DNA were measured with a NanoDrop2000 before the normalization to the required concentration (30 ng/μL). The quality control and genotyping using the Axiom^®^ Buffalo Genotyping Array 90K from Affymetrix^1^ were performed by ATLAS Biolabs GmbH (Berlin, Germany). Allele calling was carried out using Axiom Analysis Suite software V4.0.1 (Applied Biosystems by Thermo Fisher Scientific) following the pipeline for Affymetrix Axiom genotyping workflow (Nicolazzi et al., 2014) and using the last version of buffalo genome assembly (UOA_WB_1) as the reference (Low et al., 2019).

**TABLE 1:** River buffalo populations analyzed in the present study (n: samples per population).

	**Populations label**	**City**	**Country**	***n***
1	Bul_Noce	Varna	Bulgaria	58
2	Ger_Born	Born	Germany	28
3	Ger_Jüt	Jüterbog		27
4	Ger_Stad	Stadland		26
5	Ger_Wies	Wiesenburg		28
6	Hun_HT	Földes	Hungary	19
7	Hun_Csak	Csákvar		17
8	Hun_Tisz	Tiszataj		19
9	Rom_Mera	Mera	Romania	16
10	Rom_Serc	Sercaia		47
	Total number			285

### Datasets Updating and Merging

We retrieved previously reported datasets from Colli et al. (2018a) and Deng et al. (2019a) available in the Dryad Repository (Table 2).^2^ To solve the incongruity between the marker positions when merging genotypes, we retained only those SNPs that were common in the three data sets. We further removed SNPs which mapped on sexual and mitochondrial bases, as well as those with unknown chromosome coordinates to perform an autosomal analysis. The remaining panel of 36,759 SNPs was then updated to the last version of the water buffalo genome (UOA_WB_1) through the commands ***–***update-map, ***–***update-chr, –update-alleles of PLINK V1.7 software (Purcell et al., 2007). The three datasets were then merged using the merge module in PLINK. Individually, the quality control filters were applied to remove variants with a minor allele frequency lower than 0.05 or a rate of missing genotyping ***>***10%. The final dataset consisted of 36,014 SNPs and 477 individuals to perform the subsequent genetic population and structure analyses (Supplementary Table 1).

**TABLE 2:** Populations of buffalo with publicly available genotype data included in the present genome analysis (n: samples per population).

	**Populations label**	**Geographical origin**	***n***	**References**
1	Bul_Colli	Bulgaria	11	Colli et al., 2018a
2	Ita_Colli	Italy	15	
3	Mozamb	Mozambique	7	
4	Rivbr_Ana	Turkey	15	
5	Rivbr_Mur	Brazil	15	
6	RivCo	Colombia	12	
7	RivEg	Egypt	15	
8	Rivir_Aza, Rivir_Khu, Rivir_Maz	Iran	27	
9	RivPk_in_Mur	India	5	
10	RivPk_Azk, RivPk_Kun, RivPk_Nil	Pakistan	28	
11	Rom_Colli	Romania	9	
12	Ita_Deng	Italy	35	Deng et al., 2019a

### Genetic Relationship and Population Structure

A multidimensional scaling (MDS) analysis was performed to explore the genetic relationship between four different buffalo populations. To this end, a symmetric matrix of the identity-by-state (IBS) distances, for all pairs of individuals, was generated in PLINK V1.7 software (Purcell et al., 2007) based on the proportion of alleles shared and visualized using ggplot R package (Wickham, 2016). Observed (Ho) and Expected (He) heterozygosity for ten buffalo populations genotyped in this study (Bulgaria, Germany, Hungary, and Romania) were also estimated using PLINK V1.7 (Purcell et al., 2007). The maximum-likelihood approach was used to estimate the population structure through ADMIXTURE V1.3 Software (Alexander et al., 2009) with default parameters. This software modulates the probability of the observed genotypes considering the ancestry proportion and population allele frequencies. The best number of clusters (K-value) is estimated by the model as that reporting the lower cross-validation error. As an additional approach for the analysis of the population structure, a phylogenetic tree was built using the R package APE (Paradis and Schliep, 2019) based on computed Wright’s Fixation Index (F_ST_) (Wright, 1965) matrix obtained with Arlequin 3.5 (Excoffier and Lischer, 2010).

### Run of Homozygosity

Analysis of run of homozygosity (ROH) was exclusively conducted on Central and East Europe buffalo populations genotyped in this study (Table 1) and based on a panel of 61,813 autosomal SNPs available after the application of quality control filters, described above, to eliminate sexual, mitochondrial, and unknown chromosome coordinate SNPs, as well as those with a minor allele frequency lower than 0.01 and rate of missing genotyping >10%. The detection of ROH was performed using PLINK V1.7 (Purcell et al., 2007), a sliding window of 1,000 kb was designed to detect regions matching the following parameters: minimum size of 50 SNPs (–homozyg-snp 50), minimum density of SNPs of 1 SNP every 100 kb (–homozyg-density 100). We allowed one heterozygous SNP (–homozyg-window-het 1), a distance of homozygosity SNPs within the window of 250 kb (–homozyg-gap 250), and two missing SNPs (–homozyg-window-missing 2) per ROH. The identified ROH were cataloged in four categories according to its length: 1–5 Mb, 5–15 Mb, 15–30 Mb, and >30 Mb. Afterward, from ROH, we also calculated the inbreeding coefficient (F_ROH_) using R package “DetectRUN” (Biscarini et al., 2018) and applying the formula:

$$F_{\text{ROH}}=\frac{\sum L_{\text{ROH}}}{L_{\text{genome}}}$$

Σ*L*_ROH_ is the sum of the length of ROH for each individual, and *L*_genome_ is the length of the genome analyzed, which was around 2.65 Gb in buffalo.

**FIGURE 2:**
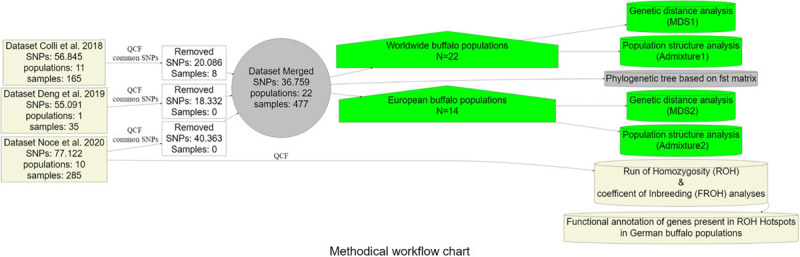
Methodical workflow chart summarizing the analysis pipeline followed in the current study. QCF = Quality control filters (MAF < 0.05, -geno 0.01, -mind 0.01).

The frequency of occurrence of SNPs into an ROH was analyzed, identifying the genomic regions most commonly associated with ROH. Regions were selected containing the top 1% of most common ROH-associated SNPs (ROH hotspots or ROH islands) (Purfield et al., 2017; Mastrangelo et al., 2018). Graphical visualization was performed using R package qqman (Turner, 2014) by constructing Manhattan plots. For the identification of genomic regions associated with ROH hotspots, we used NCBI map viewer of the water buffalo UOA_WB_1. Functional annotation was performed by the use of DAVID V6.8 (Huang et al., 2009) and PANTHER (Thomas et al., 2003) software. The analysis pipeline applied in this study is provided as a flow chart (Figure 2).

## Results

### Analysis of Genetic Diversity and Population Structure

**FIGURE 3:**
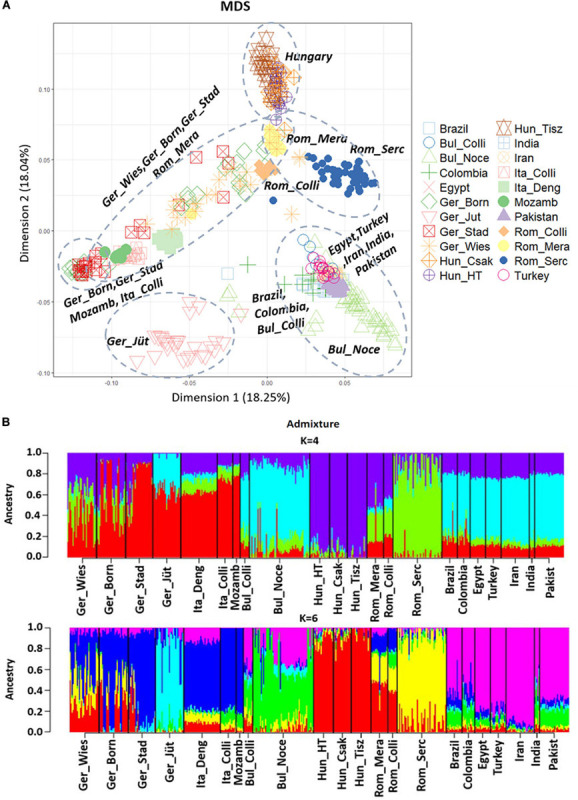
**(A)** Multi-Dimensional Scaling (MDS) plot based on genome-wide identity-by-state pairwise inferred with PLINK V1.7, including 36.014 SNPs from 477 individuals. This plot shows the genetic distance between all the 22 populations analyzed worldwide and published in part (Colli et al., 2018a; Deng et al., 2019a). The percentage of variances captured for each dimension is reported in brackets. The dashed circles mark the populations that cluster together. **(B)** Admixture analysis results at K = 4 and 6, including 22 buffalo populations worldwide. K values indicate the number of ancestries estimation (clusters), K = 6 was the best fitting solution for the lowest cross-validation error reported (CV = 0.61856). Populations are divided by a vertical black line, and it is partitioned into K colored segments that represent the population’s estimated membership fractions in K clusters. The 22 populations are localized in Germany (Ger_Wies, Wiesenburg, *n* = 28: Ger_Born, Born, *n* = 28; Ger_Stad, Stadland, *n* = 26; Ger_Jüt, Jüterbog, *n* = 27), Italy (Ita_Deng according to Deng et al., 2019a, *n* = 35; Ita_Colli according to Colli et al., 2018a, *n* = 15), Mozambique (Mozamb according to Colli et al., 2018a, *n* = 7), Bulgaria (Bul_Colli according to Colli et al., 2018a, *n* = 11), Bulgaria (Bul_Noce, *n* = 58), Hungary (Hun_HT, Földes, *n* = 19; Hun_Csak, Csákvar, *n* = 17; Hun_Tisz, Tiszataj, *n* = 19), Romania (Rom_Colli according to Colli et al., 2018a, *n* = 9; Rom_Mera, Mera, *n* = 16; Rom_Serc, Sercaia, *n* = 47), and also according to Colli et al., 2018a: Brazil (*n* = 15), Colombia (*n* = 12), Egypt (*n* = 15), Turkey (*n* = 15), Iran (*n* = 27), India (*n* = 5), and Pakistan (*n* = 28). Population labels and the number of individuals of both analyses are reported in Tables 1, 2.

**FIGURE 4:**
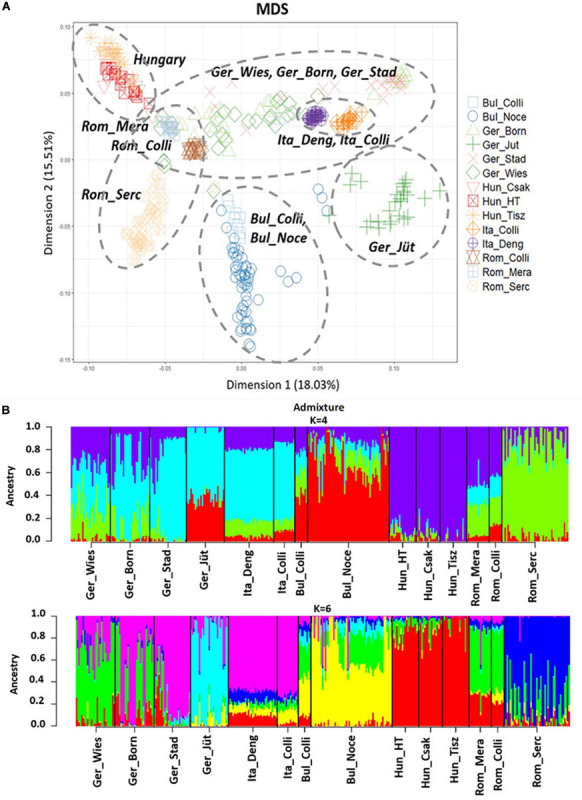
**(A)** Multidimensional scaling (MDS) plot based on European buffalo populations available in this work. The dashed circles mark the populations that cluster together. **(B)** Admixture analysis results at K = 4 and 6 exclusively in European buffalo populations. K = 6 was the best fitting solution for the lowest cross-validation error reported (CV = 0.59712). Abbreviations and sample numbers are specified in Figure 3.

The MDS plot (Figure 3A) of 22 buffalo populations (Tables 1, 2) with different geographic distributions revealed a clear separation of European buffalo from other populations distributed worldwide. Indian-Pakistan and Latino-American buffalos were presented in a single cluster together with Bulgarian Murrah buffalos as the only exception of a European population. Four different groups could be distinguished in the rest of the European population: the Hungarian cluster, which included all three farms, the Romanian cluster, which showed one farm separated from the others (Rom_Serc), the Italian cluster, which included samples from Mozambique, and a German cluster from Brandenburg (Ger_Jüt), which was separated from the other populations. The remaining animals from Germany were mixed and scattered between Romanian and Italian populations. The admixture analysis carried out on genetic information from 22 buffalo populations available with global distribution (Figure 3B) revealed a higher diversity among European populations than groups from India, Pakistan, the Middle East, and Latin America. K = 4 evidenced that the Bulgarian buffalos form the only European population that shared a high average of alleles with the non-European groups. The Romanian group showed mixed ancestry, except for Rom_Serc. Low admixture levels were found in the Hungarian group. A high admixture of buffalo was found in all German farms, noticeably with a different structure in the buffalo from Brandenburg (Ger_Jüt). In Figure 4, we aimed to focus on the relative genetic distance and structure exclusively among European populations (*N* = 14). The MDS plot (Figure 4A) showed that after excluding Mozambique buffalos, the Italian samples were presented as a more homogenous and distinct cluster. The same appeared for the two Bulgarian populations. Furthermore, animals from two German farms (Lower Saxony – Ger_Stad, Mecklenburg-Western Pomerania – Ger_Born) had relations to the Italian cluster, while animals from Saxony (Ger_Wies) appeared to be related more closely to the Romanian buffalos. Buffalos from Brandenburg (Ger_Jüt) could be positioned closer to the Bulgarian cluster. From K = 6 of admixture analysis including exclusively European populations (Figure 4B), three principal components were distinguished in Central and Southeast Europe: the Mediterranean pattern, including Italian and German populations, the Hungarian-Romanian, and the Bulgarian ones. Buffalos from farms in Germany displayed several mixed components, Lower Saxony buffalos (G_Stad) had a higher proportion of Italian Mediterranean background compared to herds in Mecklenburg-Western Pomerania (G_Born) and Saxony (G_Wies). Saxonian buffalos (G_Wies) showed a consistent portion of alleles from Romania, while the comparison at K = 4 and 6 revealed that buffalos from Brandenburg (G_Jüt) mainly contained Bulgarian genetic background.

**FIGURE 5:**
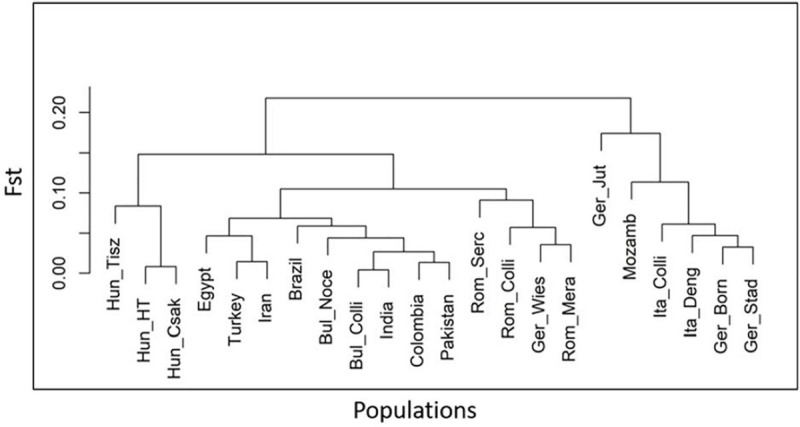
Phylogenetic tree indicating the genetic distance of 22 buffalo populations, analyzed and published in part (Colli et al., 2018a; Deng et al., 2019a), with worldwide distributions based on the fixation index (FST) matrix obtained from 36,014 SNPs in 477 individuals using Arlequine software. Abbreviations and sample numbers are specified in Figure 3.

The results of the heterozygosity metric ranged from Bulgaria to Hungary (Hung_Csak, Hung_Tisz) with 0.41–0.35 for observed values (Ho) and 0.40–0.35/0.32 for expected values (He), respectively. Similar heterozygosity values were found in the Hungarian and Romanian samples, lowest in relation to the other population groups (from 0.35 to 0.38 Ho and from 0.32 to 0.36 He), while German buffalos had the highest values of diversity, after the Bulgarian buffalo population, between groups, and within its group (Table 3). To support the interpretation of the Admixture and MDS analyses, we also measured the population differentiation due to genetic structure by an F_ST_ pairwise distance analysis of 36,014 SNPs in all individuals of 22 populations distributed worldwide. The phylogenetic tree based on the F_ST_ index matrix (Supplementary Table 2) was consistent with the Admixture analysis (Figure 5). Two principal groups defined the difference between the Italian Mediterranean and the Murrah breeds. Along with the Italian group, Mozambique and three German herds, including Mecklenburg-Western Pomerania (Ger_Born), Lower Saxony (Ger_Stad), and Brandenburg (Ger_Jüt) clustered, while Brandenburg formed a separate branch. In the second group, Murrah breeds from Pakistan and India clustered with Latino America, Middle East, and Bulgarian samples. Hungarian and Romanian buffalos stood out, while animals from Saxony (Ger_Wies) grouped with animals from Romanian clusters.

**TABLE 3:** Observed (Ho) and expected (He) heterozygosity of buffalo populations *de novo* genotyped distributed in Bulgaria, Germany, Hungary, and Romania (abbreviations are defined in Table 1).

**Populations**	**Ho**	**He**
Bul_Noce	0.41	0.40
Ger_ Born	0.39	0.39
Ger _Jüt	0.39	0.35
Ger _Stad	0.38	0.38
Ger _Wies	0.40	0.38
Hun_HT	0.37	0.34
Hun_Csak	0.35	0.35
Hun_Tisz	0.35	0.32
Rom_Mera	0.38	0.37
Rom_Serc	0.38	0.36

### Run of Homozygosity

**FIGURE 6:**
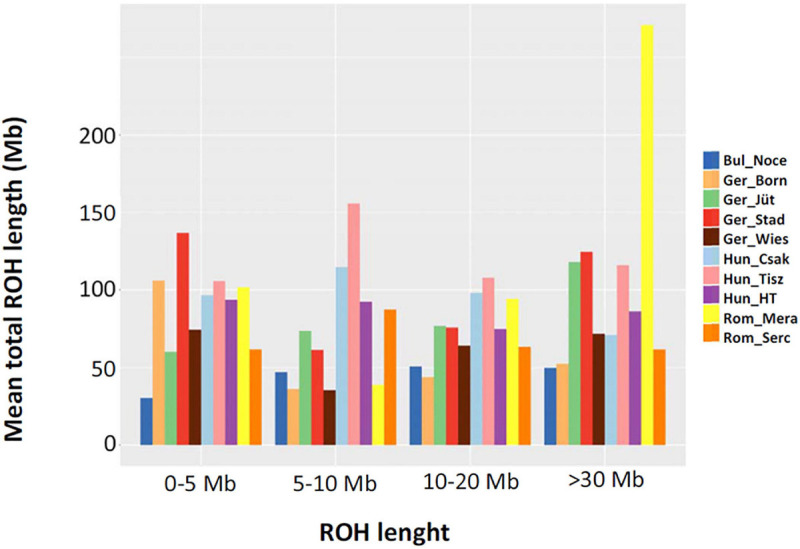
The mean sum of runs of homozygosity (ROH) per population within each ROH length category in samples collected from buffalos in Bulgaria (Bul_Noce), Romania (Rom_Mera, Rom_Serc), Hungary (Hun_Csak, Hun_Tisz, Hun_HT), and Germany (Ger_Born, Ger_Jüt, Ger_Stad, Ger_Wies). Data include 61,813 SNPs genotyped in 285 samples (Mb: megabase); all other abbreviations and sample numbers are specified in Figure 3.

**FIGURE 7:**
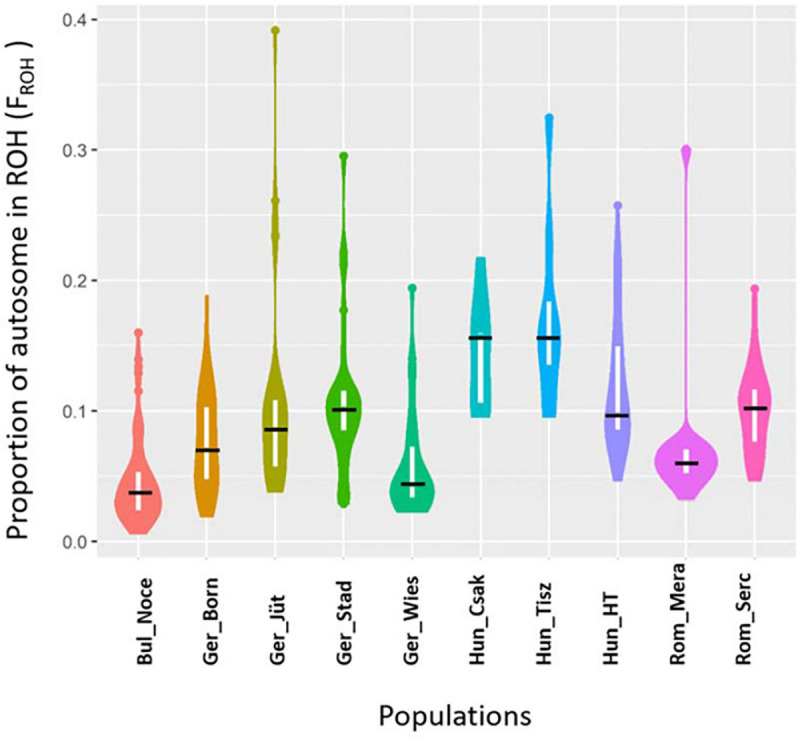
Violin Plot reporting the proportion of autosome covered in run of homozygosity (ROH) aka Genomic inbreeding coefficients (F_ROH_) in Buffalo populations from Bulgaria (Bul_Noce), Romania (Rom_Mera, Rom_Serc), Hungary (Hun_Csak, Hun_Tisz, Hun_HT), and Germany (Ger_Born, Ger_Jüt, Ger_Stad, Ger_Wies). The black line indicates the median of F_ROH_ within the breed. Abbreviations and sample numbers are specified in Figure 3.

In order to make better use of all information and to learn about the demographic past and/or recent history of the buffalo populations of Central and Southeast Europe, we performed an ROH analysis based on 285 samples genotyped in this study (Table 1), for which a panel of 61,813 autosomal SNPs was available (Figure 6). We found 10,797 ROH regions distributed in four categories (0–5, 5–15, 15–30, >30 Mb) with patterns different for each population. Among German buffalos, Ger_Born had the highest average of ROH in 0–5 Mb size category, indicative of ancient inbreeding, which decreased in all the other categories. Ger_Jüt showed the opposite trend, with the highest average in the long ROH category (>30 Mb), suggestive of the small effective population size and more recent inbreeding. The highest average of both short and long ROH categories was found in Ger_Stad. Buffalos from Saxony (Ger_Wies) showed an average of ROH quite similar in all categories except a decrease in the 5–15 Mb category. Figure 7 displays an average inbreeding coefficient calculated from the individual sum of ROH tracts per population, and in Table 4, the corresponding values are reported. The Bulgarian buffalo population had the lowest average of homozygosity in the genome (116.93 Mb) and, consequently, the lowest level of inbreeding (F_ROH_ = 0.047). Romanian samples from Mera village had 185.31 Mb of the genome in ROH with an F_ROH_ of 0.074, lower than the second farm (Rom_Serc) with 252.31 Mb and F_ROH_ 0.102. The buffalo population in Mera (Rom_Mera) is distributed in smaller herds from multiple private farms. The second Romanian population (Rom_Serc) studied here is located in Sercaia and is managed by the Institute of Research and Development for Buffalo Breeding. Buffalos from Sercaia were characterized by ancient inbreeding, showing the highest proportion of ROH in the category 5–15 Mb. These values are in agreement with the results presented in Figure 6, where both Romanian buffalo populations presented a different trend of ROH categories. Hungarian buffalos had high ROH in their genome and high inbreeding due to a small population size reflected in the different values within the farms Csákvar (Hun_Csak) and Tiszataj (Hun_Tisz) with the highest F_ROH_ (0.144 and 0.173), Földes (Hun_HT) with lower F_ROH_ (0.121). The different values of F_ROH_ obtained within the German group were an indication of the high variability between farms (Table 4).

**TABLE 4:** Averages of individuals ROH in the genome and coefficient of F_ROH_ per population.

**Populations ID**	**Geographic location**	**average sum of ROH (Mb)**	**average F_ROH_**
Bul_Noce	Bulgary	116.93	0.047
Ger_Born	Born	192.15	0.078
Ger_Jüt	Jüterbog	270.62	0.109
Ger_Stad	Stadland	272.96	0.110
Ger_Wies	Wiesenburg	146.96	0.059
Hun_Csak	Csákvar	356.91	0.144
Hun_Tisz	Tiszataj	427.16	0.173
Hun_HT	Földes	299.01	0.121
Rom_Mera	Mera	182.45	0.074
Rom_Serc	Sercaia	252.31	0.102

*ROH, run of homozygosity; F_*ROH*_, inbreeding coefficient; Mb, Megabases; all other abbreviations are explained in Table 1.*

### Localizing ROH Hotspots and Gene Set Enrichment Analyses

**FIGURE 8:**
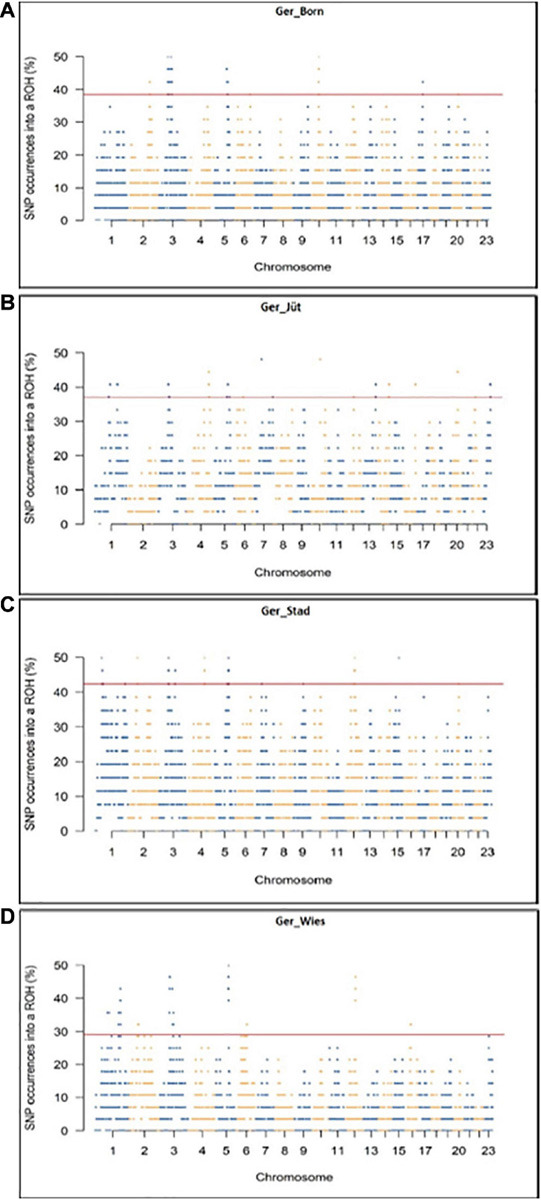
Genomic distribution of ROH islands in German buffalo samples. The x-axis represents the SNP genomic coordinate chromosome-wise, and the y-axis visualizes the frequency (%) of overlapping ROH shared among individuals. The red line indicates the threshold to define the regions containing the top 1% of most common ROH-associated SNPs (ROH hotspots or ROH islands). Populations are presented as **(A)** Mecklenburg-Western Pomerania (Ger_Born), **(B)** Brandenburg (Ger_Jüt), **(C)** Lower Saxony (Ger_Stad), and **(D)** Saxony (Ger_Wies). Abbreviations and sample numbers are specified in Figure 3.

**FIGURE 9:**
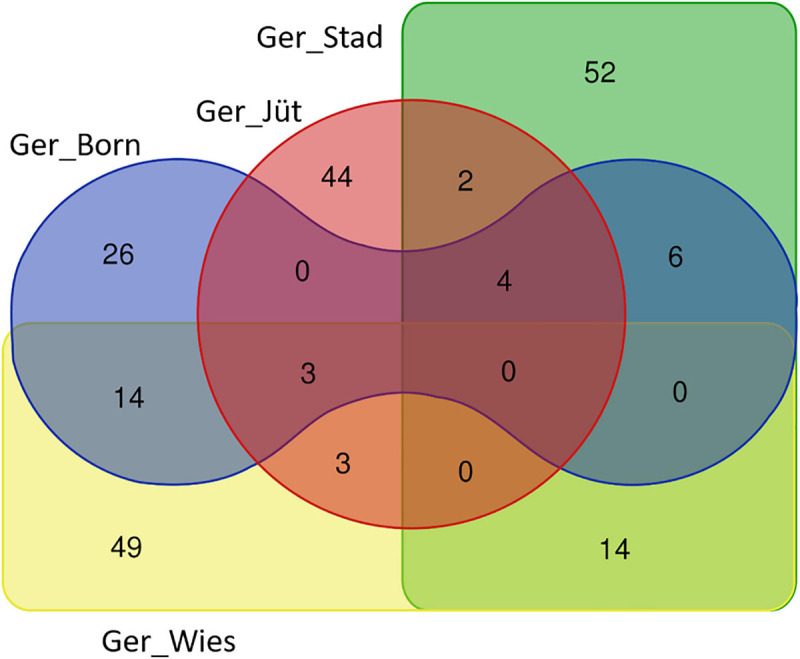
Venn diagram reporting the number of common and unique genes present in overlapping ROH regions with the highest frequency SNPs in different buffalo populations from Germany (gene names are referred by Table 6).

**FIGURE 10:**
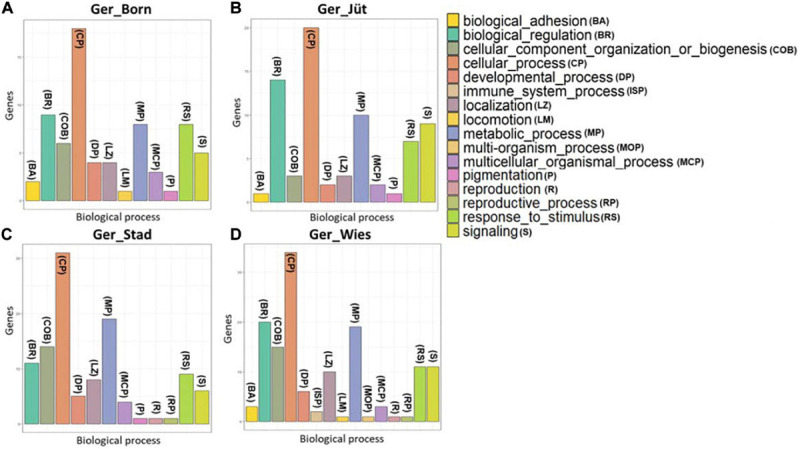
Classification of genes with hotspot SNPs in ROH genome regions. Populations are presented as **(A)** Mecklenburg-Western Pomerania (Ger_Born), **(B)** Brandenburg (Ger_Jüt), **(C)** Lower Saxony (Ger_Stad), **(D)** Saxony (Ger_Wies). Abbreviations and sample numbers are specified in Figure 3.

In order to detect the ROH hotspots, Manhattan plots were built to identify genomic regions frequently associated with ROH (Figure 8) and the SNP locus with the highest frequency (%) in those ROH (Table 5) for each German buffalo population. The thresholds to define the regions containing the top 1% of most common ROH-associated SNPs (ROH hotspots or ROH islands) were 0.38, 0.37, 0.42, and 0.29 for Mecklenburg-Western Pomerania, Brandenburg, Lower Saxony, and Saxony, respectively. With their genomic coordinates and after functional annotation (Supplementary Table 3), we were able to reveal potential candidate genes under directional selection among the different populations (Table 6 and Figure 9). We then examined genes co-localized with the ROH hotspots to identify candidate genes present in potential genomic regions that have undergone selection in each population, and where no significant (*P* ≤ 0.05) enrichment was found. All the hotspot SNPs in each population were in the short ROH length, likely suggestive of ancient inbreeding or selection events (Table 5). The populations from Brandenburg (Ger_jüt) and Lower Saxony (Ger_Stad) had similar levels of inbreeding, as also found for the populations from Mecklenburg-Western Pomerania (Ger_Born) and Saxony (Ger_Wies; Table 4). The distribution of hotspot SNPs were different in every population (Figures 8A–D), indicative of no directional selection in the groups. The highest number of genes co-localized with ROH regions were found in Saxony (83) and Lower Saxony (78), followed by Brandenburg (56) and Mecklenburg-Western Pomerania (53) (Tables 5, 6 and Figure 9) involving 11–16 biological processes (Figure 10). In particular, only in buffalos from Saxony we identified genes essential for the immune system (Il18bp, Rhog), and mammalian autophagy (Atg16l2) in chromosome 16 with 26, 21, and 15 hotspots SNPs, respectively.

**TABLE 5:** Number of significant SNPs in ROH regions, genomic distribution, and genes mapped in the same regions for each German buffalo population analyzed in this work (Chr: chomosome).

**Populations**	**Chr with hotspot SNPs**	**SNPs (total number)**	**Genes (total number)**	**ROH size range**
Ger_Born	2, 3, 5, 10, 17	679	53	0–1.4 Mb
Ger_Jüt	1, 3, 4, 5, 7, 10, 13, 14, 16, 20, 23	949	56	0–1.9 Mb
Ger_Stad	1-5, 12, 15	899	78	0–2.5 Mb
Ger_Wies	1,3, 5,6, 12, 16	635	83	0–1.4 Mb

**TABLE 6:** Shared and unique genes overlapping ROH regions with the highest frequency SNPs in the German populations.

**Populations**	**N. of Genes**	**Name of Genes**
Ger_Born,Ger_Jüt,Ger_Stad	4	RAB38, GRM5, TMEM135, CTSC
Ger_Born, Ger_Jüt, Ger_Wies	3	CA10, MFSD14B, ANXA10
Ger_Born, Ger_Stad	6	TOM1L1, MMD, LOC102416224, LOC102390200, HLF, STXBP4
Ger_Born, Ger_Wies	14	TAF1D, LOC112585128, PANX1, DEUP1, ELAVL2, SLC36A4, HEPHL1, MED17, LOC112585257, FAT3, LOC102389890, C5H11orf54, VSTM5, CEP295
Ger_Jüt, Ger_Stad	2	ME3, PRSS23
Ger_Jüt, Ger_Wies	3	NLGN1, KIF2B, NAALADL2
Ger_Stad, Ger_Wies	14	GPR17, LOC102408414, HS6ST1, AMMECR1L, PLEKHB2, UGGT1, IMP4, PTPN18, ERCC3, AMER3, LRRTM4, LOC112581251, TUBGCP5, BIN1
Ger_Born	26	FUT9, GALNTL6, MYO1B, FSTL5, LOC102389548, MFAP3L, FHL5, CAVIN2, NDUFAF4, MMS22L, MTNR1B, TMEFF2, LOC102389468, UFL1, PALLD, AADAT, CBR4, NEK1, MANEA, LOC102409600, SH3RF1, GPR63, KLHL32, CLCN3, NABP1, TMEM100
Ger_Jüt	44	NSMCE3, FAM189A1, ZNF25, ZPLD1, DIAPH3, ZNF37A, BMS1, LOC102392518, LOC112581575, TJP1, BTBD3, SIM1, RASGEF1A, LOC112581582, APBA2, FXYD4, FZD4, TASP1, ZNF248, LOC102406297, C23H10orf143, SPTLC3, GRIK2, MCHR2, GLRX3, MGMT, LOC102407296, ASCC3, LOC102400568, TARSL2, BICC1, SNRPA1, OTUD7A, CSGALNACT2, RET, LOC112578510, EBF3, PCSK6, HNRNPF, CHRM3, LOC102395743, ISM1, TM2D3, LOC102397911
Ger_Stad	52	SLC6A15, CPA6, MYBL1, AHSA2P, TYR, CCT4, VPS54, LOC102392047, PPP1R42, C15H8orf34, MGAT4C, LRRIQ1, CSMD1, SULF1, DNAJC5B, DLG2, VCPIP1, NTS, PREX2, TCF24, FAM161A, CSPP1, ARFGEF1, PDE7A, MYOM2, ARHGEF10, COPS5, WDPCP, CLN8, COMMD1, VXN, CRH, UGP2, OCA2, EHBP1, MCMDC2, ADHFE1, TSPAN19, TRIM55, KBTBD11, LOC112577753, DLGAP2, OTX1, MDH1, ALX1, B3GNT2, RRS1, NIPA1/2, ERICH1, TMEM17, RASSF9, LOC102407794
Ger_Wies	49	NIPA1, CLPB, NAB1, ART1, PGAP2, LOC102413639, CADM2, CHRNA10, NEMP2, ARHGEF4, LAMTOR1, FCHSD2, NUP98, RNF121, STIM1, PDE2A, SAP130, MFSD6, LOC112577983, ARHGEF17, PHOX2A, LOC102407599, ATG16L2, LIMS2, IWS1, WDR33, MYO7B, RELT, CCDC115, STARD10, ARAP1, SFT2D3, P2RY6, TRIM21, LOC102390798, FAM168B, P2RY2, POLR2D, RHOG, IL18BP, FOLR2/INPPL1, RRM1, CYFIP1, MAP3K2, TRIM68, PROC, ANAPC15, NUMA1, IZUMO3

## Discussion

### European Buffalos Stand Out From Others

This is the first study aimed to explore the genetic background of German buffalos in relation to other European populations (Colli et al., 2018a; Deng et al., 2019a). Our knowledge about the genetic relationships of European breeds, especially in Central Europe and Germany are still poor. A recent study from Colli et al. (2018b) reported a high genetic variability within Eastern European populations and identified recognizable traces of Indo-Pakistani background in the genome of Mediterranean buffalo (Colli et al., 2018b). Here, we compared 22 populations worldwide. The separation between Murrah (India-Pakistan, Latino America, and the Middle East) and Mediterranean (European populations) breeds was clearly evident. European buffalo populations are characterized by higher genetic diversity than the Murrah breeds, which is related to their shorter distance from the place of domestication. This could be a consequence of the natural buffalo repopulation in Europe after the Ice Age or by the distribution of domesticated buffalo by different routes (Bartosiewicz, 1999). Admixture analysis revealed that European and non-European populations do not share the same gene pool. Only Bulgarian Murrah buffalos were profoundly different from the other European animals. In K = 4, a large amount of alleles were common with Murrah breeds from India-Pakistan but not in K = 6 (Figure 3B), indicating the development of a different genetic background. In fact, the values of observed and expected heterozygosity (He = 0.40) were comparable to those previously reported by Colli et al. (2018b) in Murrah breeds from India-Pakistan, underlining their similarity and indicating an isolate-breaking effect, the mixing of previously isolated populations. From the data provided here, we have strong evidence that European buffalos stand out from other buffalo populations worldwide. We may thus assume ancient origins of European buffalos (excluding Bulgaria), supporting the idea of Krawczynski (2010) postulating that buffalos did not wholly disappear from the European continent during the late Pleistocene.

### Different Genotypes in European Buffalos and a Novel One in Hungary

Four clear clusters could be distinguished in the MDS plot when including samples from five European countries only, representing the geographical distribution of Bulgarian, Hungarian, Romanian, and the Italian population. German populations, however, were mixed and located in between the Italian and Romanian groups. In the Romanian cluster, samples collected from the National breeding facility (Rom_Serc) were separated from the other farm located in a former Hungarian village (Mera), now part of Romania. The origin of Romanian buffalo is still unclear. According to Borghese, it is a Mediterranean type adapted to the cold climate and local environment and is classified as a Mediterranean Carpathian breed (Borghese, 2005). Because of the massive decrease of their population size during the last 20 years, they are nowadays in an endangered status, distributed in small farms in some villages, and almost 98% in Transylvania used for local food production and draft strength (Matiuti et al., 2020). Our results revealed that Mera buffalos (Rom_Mera), which were distributed in multiple smaller familiar farms, were characterized by a mixture of the Italian Mediterranean, Hungarian, and Bulgarian genetic background, while buffalos from the National breeding facility (Rom_Serc) have a defined genotype. In this national breed, a higher level of F_ROH_ was found than in Mera buffalos. Moreover, the Mera herds (0.074) showed the highest proportion of long size ROH that confirms the small population size but is also indicative of recent inbreeding events. It may be due to the application of selection programs and the sign of environmental adaptation of the Mediterranean Carpathian buffalo in Rom_Serc population, while in Mera the animals are less subjected to predefined or joint selection programs. According to Karpati (1997), Mediterranean buffalos were introduced to Hungary, by the Turks, during the 16th century. The animals are distributed in small private farms used for family husbandry but mainly kept in national parks as a protected reserve (Borghese, 2005). In the MDS plot, the Hungarian cluster, including three populations (Földes, Csákvar, and Tiszataj), showed a lower admixture with Balkan populations (Figure 4B). The comparably high level of inbreeding coefficient and a high proportion of ROH, especially in short length, is indicative of ancient inbreeding events in Hungarian buffalos. Notably, the genetic background in Hungarian buffalo is comparably homogenous compared to all genotypes published before or presented here and may represent a valuable resource for local economy or breeding programs worldwide in the future. Clearly, and maybe as a surprise, the genetic background in Hungarian buffalo is different from the population in Mera, which represents a former Hungarian village. In all three Hungarian farms included in this study, the buffalos are used for meat production only and serve particularly for the establishment of local marketing together with other regional products. Under current natural breeding, there is no selection for milk production at present in the herds included, which may explain the lack of recent inbreeding. Bulgarian Murrah buffalos, instead, had the lowest proportion of ROH in each length category and the lowest F_ROH_ values between Central and East European populations. Indeed, the Bulgarian is the biggest population analyzed in this study, and the samples were collected from two different locations, which also could be related to comparably low levels of inbreeding. In the German populations, genomic relationships with Bulgarian Murrah, Italian Mediterranean breeds, and Romanian genetics were identified. Buffalos in Germany had the highest observed and expected heterozygosity values within all Central Eastern European populations studied here. The observed genetic diversity is a significant factor for their potential adaptation to the local environment and provides a valuable substrate for an upcoming selection program. Drift rather than mutation has likely created this variability due to the short period in generations and the relatively small effective population sizes (Wang, 2005). Buffalos from Brandenburg (Ger_Jüt) stood out from all other German populations studied here. As the only German population, clear genetic relations to Bulgarian buffalos can be postulated for the Jüterbog herd. In fact, part of the Bulgarian Murrah’s gene pool that was visible in the admixture at K = 4, and disappeared at K = 6, is likely an effect of genetic drift after isolation over time. The Brandenburg buffalo herd showed the highest deviation of heterozygosity values, with He being lower than Ho, suggesting Hardy-Weinberg disequilibrium and inbreeding (Mayo, 2008). The higher values of inbreeding with an increasing trend of ROH average from short to long size may further suggest recent inbreeding and isolation without any admixture since the introduction of the buffalos in Germany.

### Overlapping ROH Signals in German Buffalo Genome

The control of inbreeding in a population is necessary to avoid depression of phenotype traits (Ouborg et al., 2010; Fontanesi et al., 2015). Considering that ROH regions are not randomly present in the genome (McQuillan et al., 2008), the detection of the genomic regions frequently covered with ROH is useful. This helps to understand whether they influence any quantitative trait, especially in a population of small population size, such as local breeds (Biscarini et al., 2015). The negative effect of inbreeding depression in milk production has been reported in Egyptian (Khattab and Kawthar, 2007), South Iranian (Mirhabibi et al., 2007), and Brazilian buffalo populations (Santana et al., 2011). We found several differences among German buffalo populations in ROH analysis in terms of the number of hotspot SNPs, candidate genes in the same genomic coordinates, and biological processes in which they could be involved. In the Brandenburg (Ger_Jüt) population, the most distinguished, with a high level of inbreeding, we found the highest number of hotspot SNPs (949), distributed over 11 chromosomes and concerned only 57 genes involved in 11 biological processes. While Saxony buffalos (Ger_Wies), the population with the lowest inbreeding level and the lowest number of hotspot SNPs (635), showed their distribution limited to 6 chromosomes but to 83 genes involved in 15 biological processes. Overlapping ROH signals affecting genes involved in the immune system (*Il18bp, Rhog*) and autophagy (*Atg16l2*) were only found in Saxony. It is reported that the gene Atg16l2 (autophagy related 16 like 2) influences the adaptation of the immune system to the recovery from mastitis in Danish Holstein cattle (Welderufael et al., 2018). From these very initial interpretations of the potential effects of local selection for functional traits, we may get an idea of how genomic selection could be used to improve animal health and performance in the future. Certainly, additional studies of gene expression, metabolism, or animal health are needed to link SNP markers with particular phenotypes. We are aware of the limitations of our study. Only four different farms in Germany are included that represent less than 15% of the German buffalo population. Moreover, the selection of farms in Eastern Europe was based on potential familiar relationships and can be seen as a start only. For German and other European countries, higher coverage, including more herds and additional countries to further exploit the full setting of genetic diversity in Europe, and an in-depth analysis of signatures of past selection combining several metrics together with ROH is required. Finally, in the genotyping array designed for buffalo, only 30% of SNPs from European breeds (Italian Mediterranean) have been included, which could be a further limitation to obtain genomic information about European buffalo populations. Accordingly, we may initiate new genome sequencing initiatives to develop more informative SNP arrays to improve further functional genome analysis and genome-based selection in European buffalo.

## Conclusion

This work aimed to contribute to an improved understanding of Central and Southeast European buffalo populations from a genomic perspective. We defined the genetic characteristics of European buffalo populations in Romania, Bulgaria, and Hungary. Together with the Italian Mediterranean breed, these populations contribute to the high degree of genetic diversity of European buffalos. Incidentally, we may have genetic evidence for a novel or original Hungarian breed, characterized by outmost uniformity in three different farms with no clear relations to any other genotype described so far. Since all farms are in the close neighborhood, follow-on studies may help to unravel the origin of the Hungarian population. This novel genetic identity found in Hungarian buffalos thus adds one additional specific breed to the existing genetic diversity in Europe, which so far consisted only of the Italian Mediterranean, Bulgarian Murrah, and Romanian genetics.

This is the first genetic characterization of buffalos in Germany, where farming started only about 40 years ago and therefore is in its infancy. We have identified familiar relations to all genotypes identified in European buffalos so far with the exception of Hungary. Importantly and based only on a comparably small number of herds, a diverse genetic make-up is available in European and German buffalos, which can be seen as an excellent prerequisite for the development of Buffalo-based bioeconomy in Europe based on local breeds characterized by unique features.

## Data Availability Statement

Our data set has been uploaded in the Dryad Repository, https://datadryad.org/stash/share/eT_rs3441UHCMlN4qpbRi8u6sFPHdX-ZtDPuVS5TpSI and https://datadryad.org/resource/doi:10.5061/dryad.h0cc7.

## Ethics Statement

Ethical review and approval was not required for the animal study because sampling occurred as part of livestock breeding programs which are granted by international law and therefore are not reviewed by ethical committees. Written informed consent was obtained from the owners for the participation of their animals in this study.

## Author Contributions

AN, KW, and AH conceived the project and designed the experiments. AN performed the laboratory part. AN, SQ, RG-P, and ML-S performed the data analysis. AN, SQ, RG-B, JB, RK, and AH contributed to the interpretation of results and wrote the manuscript. MiT, M-AF, HP, AB, LV, CH, LH, PP, YI, TP, WL, MaT, and AH collected or provided samples and contributed to data production. All authors read, made corrections, and approved the final version of the manuscript.

## Conflict of Interest

M-AF, MiT, HP, and RK are employed by the companies Gut Darss GmbH & Co., Hof am Meer GmBH, Wiesenburger Land eG, and Energiequelle GmbH, respectively. The remaining authors declare that the research was conducted in the absence of any commercial or financial relationships that could be construed as a potential conflict of interest.

## Footnotes

1 http://www.affymetrix.com

2 https://datadryad.org/resource/doi:10.5061/dryad.h0cc7; https://doi.org/10.5061/dryad.310pf05

## Supplementary Material

Supplementary Table 1 A descriptive summary of three SNP panels used in this study.

Supplementary Table 2 Wright’s Fixation Index (FST) matrix computed with 36,014 SNPs in 22 buffalo populations.

Supplementary Table 3 List of potential candidate genes and relative functional annotation found in ROH hotspots region in German buffalo populations: **(a)** Mecklenburg-Western Pomerania, **(b)** Brandenburg, **(c)** Lower Saxony, **(d)** Saxony.
