# Supplementary material for: Genetic Diversity of Bubalus bubalis in Germany and Global Relations of Its Genetic Background
Source: Front Genet. 2021 Jan 22;11:610353. doi: 10.3389/fgene.2020.610353 (PMC7863760; doi:10.3389/fgene.2020.610353)
Supplement: Supplementary Table 1 — A descriptive summary of three SNP panels used in this study. [file Table_1.DOCX]

| Table S1: A descriptive summary of three SNP panels used in this study. | | | |
| --- | --- | --- | --- |
|  | **Colli et al. 2018** | **Deng et al. 2019** | **Own data** |
| N. genotyped animals | 165 | 35 | 285 |
| Tot. Number SNP before merge | 56.845 | 55.091 | 77.122 |
| SNPs without chr. coordinate | 6.119 | 0 | 9.324 |
| SNPs in sex chr. and mitochondrial | 2.133 | 0 | 2.144 |
| Tot. removed SNP | 20.086 | 18.332 | 40.363 |
| Samples Filters (–mind 0,1) | 8 | 0 | 0 |
| Total samples after quality controls | 157 | 35 | 285 |
| SNPs in common used for merge | 36.759 | | |
| Quality control Threshold filter (–geno 0,1) | 673 | | |
| Quality control Threshold filter (–maf 0,05) | 72 | | |
| **SNPs available for analysis in the final dataset** | **36.014** | | |
